# Nanoneedle-Based Transdermal Gene Delivery: A Minimally Invasive Strategy for Gene Therapy

**DOI:** 10.3390/ijms26136235

**Published:** 2025-06-27

**Authors:** Fatma Julide Akbuğa, Muhammet Davut Arpa, Emine Şalva

**Affiliations:** 1 Department of Pharmaceutical Technology, School of Pharmacy, Istanbul Medipol University, 34815 İstanbul, Türkiye; mdarpa@medipol.edu.tr; 2 Department of Pharmaceutical Biotechnology, Faculty of Pharmacy, Inonu University, 44280 Malatya, Türkiye; emine.salva@inonu.edu.tr

**Keywords:** nanoneedle, gene delivery, transdermal, siRNA, DNA, CRISPR

## Abstract

Transdermal drug delivery systems have recently been explored as an alternative to oral systems, which have many challenges. Due to the limitations of first-generation transdermal systems, second- and third-generation systems have been developed, among which microneedles have been the most remarkable products. Building on the advancements of nanotechnology, nanoneedles have recently been developed. Gene therapy molecules—such as DNA, RNA, siRNA, miRNA, and other nucleic acids—are typically delivered using viral or chemical carriers, but these methods face several challenges. In this context, nanoneedles offer a promising and efficient solution for delivering these large molecules. Nanoneedles are a biocompatible and reliable physical method for gene delivery, enabling transdermal administration by penetrating the skin barrier and delivering nucleic acids directly into cells. Their ability to penetrate cellular barriers with minimal invasiveness makes them advantageous for delivering genetic materials. This review will focus on the potential applications of nanoneedles in pharmaceutical contexts, especially in gene therapy. In addition, information on the properties, structure, and fabrication of nanoneedles is also provided.

## 1. Introduction

The process of treating disease by introducing genetic material into cells is known as gene therapy [1]. This therapy involves either the addition of new copies of genes to replace damaged ones or the correction of defective or missing genes with healthy versions [2]. To date, the U.S. Food and Drug Administration (FDA) has approved 21 nucleic acid-based medicines [3]. However, several challenges—such as the inability to target diseased tissues and cells precisely, the rapid clearance of genes from the circulation, and the degradation of genes—limit the success of gene therapy.

For gene therapy to be effective, the genetic material must be delivered to the target cells through an appropriate method. Biological (viral), chemical, and physical techniques are employed for this purpose [4], but each approach presents distinct challenges, and no current gene delivery method is entirely free from problem.

**Figure 1:**
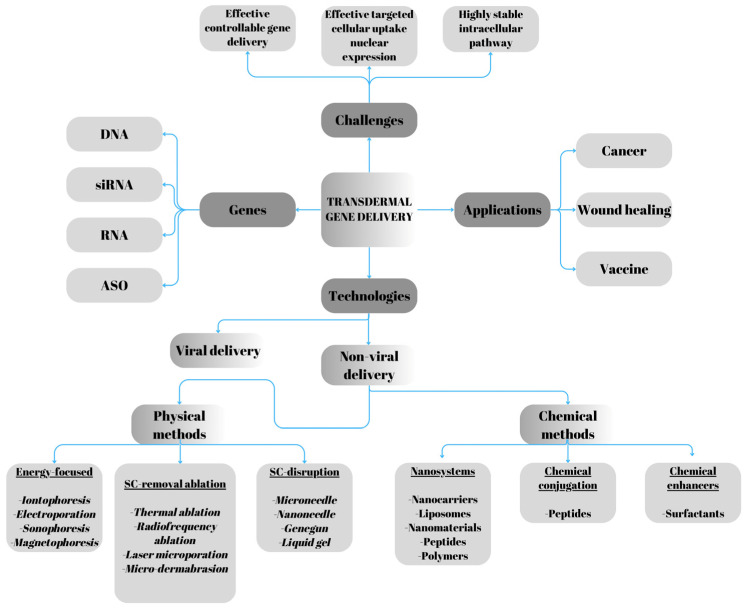
Challenges, applications, and technologies in transdermal gene delivery. Adapted from Chen’s review [15].

Viral vectors remain the most effective tools for intracellular gene delivery. However, they pose limitations, including low-gene-delivery capacity, mutagenicity, and particularly immunogenicity [5]. In contrast, non-viral strategies offer several advantages, such as localized gene expression, improved safety profiles, the capacity to carry genes of all sizes, and lower production costs. Nonetheless, these systems still present obstacles to effective gene therapy. After entering the target cell, DNA must dissociate from the carrier vector, escape from endosomes, and cross the nuclear membrane. Therefore, optimizing the efficiency and specificity of gene delivery remains critical [6]. Non-viral gene delivery methods can be broadly classified into two categories: physical and chemical approaches. Physical techniques include electroporation [7], microinjection [8], ultrasound [9], and laser treatment [10]. These methods create temporary pores in the cell membrane, facilitating the entry of drugs into the cytoplasm. Chemical methods, on the other hand, involve agents that encapsulate the genetic material or form complexes with it. Molecules such as DNA and siRNA enter cells via conjugation or encapsulation [11,12,13,14]. Recent studies have explored the potential of nanoparticle-based non-viral vectors for gene delivery in clinical applications, revealing a growing number of clinical trials employing non-viral carriers. Notably, promising results have been achieved with lipid- and polymer-based delivery systems. Additionally, gene editing has emerged as a powerful tool for modifying genes within cells, employing advanced techniques such as Zinc Finger Nucleases (ZFNs), Transcription Activator-Like Effector Nucleases (TALENs), and the CRISPR-Cas system. Nanoneedles offer a promising platform for the direct intracellular delivery of these genome-editing tools, enabling precise, efficient, and minimally invasive genetic manipulation [5]. The key applications, technologies, and challenges associated with transdermal gene delivery are summarized in Figure 1.

A recently developed method of gene transfer employs the use of nanoneedles to insert genetic material directly into the cell cytosol. These nanoneedles, with a diameter of less than 200 nm, are operated using atomic force microscopy (AFM), which enables their forcible insertion into cells by applying a precise force to penetrate the cell membrane and deliver the payload intracellularly. The biomolecules delivered through nanoneedles, such as siRNA and peptides, exhibit biological activity and can effectively transport molecules into the cytosol or the nucleus (in the case of DNA) [16].

Nanoneedles are manufactured through processes similar to those used for microneedles and are prepared using comparable techniques. However, they cause significantly less skin damage than microneedles. The challenge of delivering drugs transdermally arises from the unique physiology of the skin. The outermost layer of the epidermis, the *stratum corneum*, is composed of keratinized, intact proteinaceous corneocytes arranged in two layers and embedded in an extracellular lipid matrix. This structure forms a formidable barrier that limits drug penetration and absorption [17]. The lipophilic nature of the *stratum corneum* further restricts the permeation of therapeutic agents, particularly charged, high-molecular-weight, and hydrophilic substances such as peptides, DNA, and small interfering RNA (siRNA) [18]. Additionally, the physicochemical properties of the drug significantly impact the success of transdermal delivery. Transdermal drug delivery offers several advantages, including improved patient compliance and the use of a larger surface area of the skin for rapid drug administration. This approach also circumvents the low oral bioavailability often associated with biological agents due to first-pass metabolism, and it provides pain relief compared to traditional injections. However, despite the widespread use of topical delivery methods, the effective administration of biological agents, including nucleic acids, proteins, and peptides, remains challenging due to the skin’s robust barrier function. To overcome this barrier, microporation techniques have been developed to create micron-sized pores in the upper layers of the skin, facilitating the targeted delivery of drugs and macromolecules. Several methods are employed to create microchannels, including thermal ablation, radiofrequency, electroporation, ultrasound, lasers, high-pressure jets, and microneedles. These techniques enable the disruption of the skin barrier, providing additional driving force for drug transport [19,20]. Beyond drug delivery, these methods are also utilized for minimally invasive assays and monitoring of biological fluids. While microneedles offer numerous advantages, their repeated use may cause skin irritation. Nanoneedles, with their smaller size, may mitigate this issue and prove more effective for prolonged use [21]. However, research involving nanoneedles as drug delivery systems remains limited compared to microneedles (as summarized in Table 1). This review provides an overview of nanoneedles, discussing their properties, production methods, and associated challenges. It also examines recent studies on the use of nanoneedles in gene therapy and explores the future directions for research in this field.

## 2. Nanoneedles

The terminology used to describe nanoneedle structures varies widely and is often inconsistent. These structures may be referred to as nanowires, nanorods, nanofibers, nanotubes, nanopillars, and more. They are characterized by a high aspect ratio (the ratio of length to diameter), a defining feature among these types of structures. Nanoneedles have a broad range of potential applications, including drug delivery, the extraction of cellular contents, and the measurement of electrochemical signals. Notably, they can perform all three functions simultaneously [30]. Nanoneedles offer a promising alternative for drug delivery, as other topical methods often require high doses, increasing the risk of adverse effects, necessitating frequent applications, and resulting in low drug bioavailability. By penetrating cells, nanoneedles enable highly efficient gene transfer. A study by Han et al. [31] reported that nanoneedles achieved a gene transfer efficiency of over 70% in human mesenchymal stem cells by delivering genetic material directly into the nucleus without causing significant harm to the cells. However, the precise mechanism of intracellular transport remains debated, with other studies suggesting alternative endocytic pathways.

## 3. Advantages of Nanoneedles

Nanoneedles (NNs) represent a novel technology capable of controlling drug release. Intracellular Drug Delivery: NNs can be used to deliver large molecules, such as nucleic acids, proteins, peptides, and even cells, directly into the cytoplasm. Pain-Free Delivery: The extremely small diameter of nanoneedles allows them to penetrate the skin without disturbing nerve endings in the dermis, enabling painless drug administration. Targeted Delivery: NNs reduce the accumulation of drugs at non-target sites, making them ideal for delivering high-potency drugs. Additionally, they can facilitate the targeting of antigens to antigen-presenting cells in the skin, further enhancing their application in immunotherapy. Delivery of Toxic Drugs: By ensuring precise delivery to intended targets, nanoneedles minimize side effects and enhance the therapeutic outcome of toxic drugs. Their targeted delivery system reduces unwanted side effects, achieving greater therapeutic efficiency. Diverse Applications: Nanoneedles are suitable for various applications, including ocular drug delivery and vaccine administration. When employed for vaccines, they offer a painless, effective method of treatment. Cancer Therapy: Nanoneedles are increasingly recognized as a highly effective drug delivery system for cancer therapy, facilitating the precise delivery of anticancer drugs to specific sites within the body [32].

Due to their ability to interact directly with the cytoplasm, nanoneedles have become a powerful tool for studying living cells with minimal disruption to cellular functions. NNs can be applied as either mobile or immobile systems. Mobile nanoneedles: These free-flowing structures can be loaded with therapeutic molecules for use as particulate drug delivery systems. For example, Wu et al. demonstrated the delivery of highly drug-loaded nanoneedles containing 10-hydroxy camptothecin, achieving higher delivery efficiency with nanoneedles of higher aspect ratios [33]. Immobile nanoneedles: These are anchored to substrates, such as nanofiber networks, pads, or patches. Nanoneedle arrays, created through photolithographic patterning and deep reactive ion etching techniques [34], can be applied to cell membranes to deliver biomolecules or facilitate intracellular actions. Recent research by Chiappini [35] explored immobilized nanoneedles as biosensors for detecting intracellular processes and biomolecules. His work emphasized how the properties of nanoneedles influence biosensor performance and the disruption of cell membranes.

## 4. Disadvantages of Nanoneedles

Despite their potential, nanoneedles present several challenges. One major difficulty lies in achieving efficient delivery of biomolecules into cells. For example, Chiappini et al. [36] reported that biodegradable porous nanoneedles successfully delivered nucleic acids intracellularly and induced localized neovascularization in vivo, suggesting their potential in targeted applications. However, this localized effect may also indicate a limitation in terms of broad tissue distribution, especially when large-area or systemic delivery is required. Furthermore, Elnathan et al. [37] showed that while vertical nanowire arrays can transfect up to 90% of HEK293 cells, efficiency dropped below 60% for HeLa cells, reflecting cell-type specificity. An important consideration is the need to monitor continuous drug release for specific diseases, such as cancer, diabetes, or epilepsy, using nanoneedle systems integrated with mobile devices. Ensuring precise, sustained delivery in real time presents a significant technical challenge. Furthermore, the clinical translation of nanoneedle technology remains a major hurdle, requiring further advancements to facilitate their safe and effective application in healthcare [22]. Large-scale manufacturing remains complex and costly due to the reliance on advanced nanofabrication techniques such as electron beam lithography [24]. As reported by Kim et al. [34], conventional rigid nanoneedle arrays may struggle to conform to curved or mobile skin surfaces, potentially leading to inconsistent contact and reduced delivery efficiency. To address this, flexible elastomer patches have been developed to improve mechanical stability and surface adaptability for transdermal applications. Moreover, regulatory approval and consistent needle production are some of the issues that still need to be resolved [38].

## 5. Types of Nanoneedles

**Figure 2:**
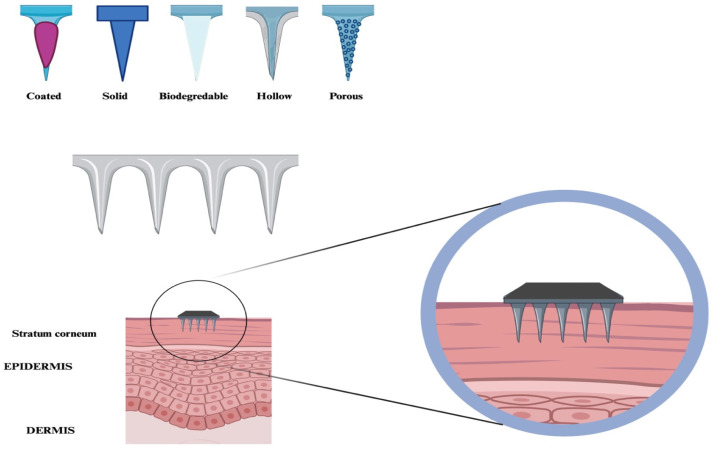
Nanoneedles are classified into different categories, including coated, solid, biodegradable, porous, and hollow. These are designed to be inserted into the *stratum corneum*.

Nanoneedles, used for delivering molecules such as nucleic acids and serving as carriers for biological probes that penetrate intracellular spaces, can be categorized into various forms, including solid, porous, hollow, and biodegradable structures (Figure 2, Table 2).

### 5.1. Solid Nanoneedles

The simplest type of nanoneedle is the solid nanoneedle structure. These are primarily used for skin pretreatment to enhance overall skin permeability. Solid nanoneedles contain drugs uniformly mixed within a polymer solution, releasing the drug upon contact with the skin. Solid silicone nanoneedles have been fabricated using the vapor–liquid–solid (VLS) method or focused ion beam (FIB) techniques. Drugs are loaded onto their surfaces through physical adsorption [16,31,45]. Electrostatic interactions are commonly used to enhance physisorption by ensuring that the surface charge of the nanoneedles is opposite to that of the adsorbed substances [16].

A typical approach involves oxidizing the silicone surface at a physiological pH, resulting in a hydrophilic, negatively charged surface [40]. Surface functionalization with amine-terminated silane—most commonly 3-(aminopropyl) triethoxysilane (APTES)—creates an electrostatically positive charge, facilitating the adsorption of proteins and nucleic acids. This positively charged surface is often preferred over negatively charged alternatives for improved molecular adsorption [40,45].

The chemical adsorption of substances onto the walls of nanoneedles has been explored; however, despite the successful loading of molecules, effective drug release was not observed [46,47]. When drugs were loaded using surface physisorption techniques, only time-limited drug application was achieved, as the drug quickly detached from the nanoneedle and diffused into the solution [16].

To address this issue, nanoneedles are coated with thick layers to prevent rapid desorption. Given the inherent complexity of nucleic acid delivery, successful delivery can be achieved by employing appropriate coated flat surfaces. Notably, the nanoneedle array does not need to function differently from flat surfaces in terms of drug delivery. The diameter of the nanoneedle tip plays a crucial role in delivery efficiency. Sharper nanoneedle tips have been shown to enhance drug delivery efficiency while simultaneously reducing cytotoxicity [48].

### 5.2. Porous Nanoneedles

Porous silicon, a biodegradable material suitable for microfabrication, possesses favorable toxicological properties and versatile transport capabilities for systemic delivery and the development of implantable systems. Porous nanoneedles can be fabricated using a combination of metal-assisted chemical etching and standard microfabrication techniques. Porous nanoneedles offer several advantages over solid nanoneedles. Their large surface area and pore volume provide an optimal reservoir for drug loading and significantly increase the charge density of the nanoneedle structure [49]. The ability of porous structures to retain drugs enables high loading concentrations. In addition to electrostatic charging, drugs prepared from molten powders can also be loaded into porous structures [50].

The pores of these nanoneedles can be sealed to protect the drug from environmental exposure, preventing premature release and allowing for controlled modulation. For example, the use of agarose to plug the pores can protect protein payloads from protease degradation without altering the release profile. A suitable coating ensures minimal drug leakage in extracellular environments with low pH, promoting maximal intracellular distribution [16].

Porous silicon also influences drug solubility by limiting diffusion through the pores and controlling the gradual dissolution and desorption of the drug [49]. Unlike solid nanoneedles, mesoporous nanoneedles can retain nanoparticles within their structure and gradually release them over time [40]. By modulating the solution properties, the rate of drug release can be finely tuned, overcoming the limitations of the rapid release profiles associated with solid nanoneedles. This approach ensures a sustained release, combining the benefits of both porous and hollow nanoneedles. However, porous nanoneedles have a limited drug reservoir, which restricts the total amount of drug that can be released. In comparison, hollow nanoneedles act as conduits, connecting to larger external reservoirs for extended drug delivery [16].

### 5.3. Hollow Nanoneedles

Microfabrication offers straightforward methods for creating hollow nanoneedles. This technique has long been used to connect cells with drug reservoirs, facilitating continuous or repeated drug delivery [41]. Hollow nanoneedles, also known as nanostraws, have been employed not only for drug delivery but also for extracting intracellular proteins and mRNA from cells [51,52,53].

Although hollow microneedles have been extensively studied [11], their relatively large size limits their use for cell analysis without causing damage. To overcome this challenge, nanoneedles based on atomic force microscopy (AFM) were developed, enabling single-cell applications [54,55].

The fabrication of hollow nanoneedles begins with the formation of nanopores, which are coated with a thin dielectric film. This film is then selectively etched—retreating from horizontal surfaces while remaining on the pore walls as a lining shell. Further selective etching of the surrounding material creates the hollow structure. This method improves coverage compared to earlier techniques but results in nanoneedles with larger diameters, requiring surfactants to assist drug delivery. However, the limited aspect ratios and larger sizes of these needles present challenges, including compromised biocompatibility [16].

Hollow nanoneedles function as conduits that connect the cell cytosol to drug reservoirs, without being pre-loaded with medication [41]. These nanoneedles resemble conventional needles and microneedles, serving as channels that deliver drugs from large external reservoirs at high concentrations. However, diffusion-based transport is relatively slow. Nanoneedles are often arranged in arrays, which feed from a common reservoir to achieve higher throughput compared to manual delivery, and although similar to microneedles, these channels can become blocked by accumulated proteins, limiting their long-term usability [41,56].

Several studies have reported the fabrication of ordered hollow nanoneedles from silicon-on-insulator (SOI) [41] and silicon wafers [57] using electron beam (EB) writing. While these techniques have successfully produced nanostructures, EB lithography faces challenges related to cost and scalability for large-scale manufacturing [58].

Stepper lithography offers a potential solution to these limitations by improving production volume, making it a suitable method for high-throughput nanoneedle fabrication [59,60]. However, stepper lithography, despite its higher throughput, provides a less precise resolution than EB lithography, and the fabrication of complex hollow nanoneedle architectures using this method remains challenging [60]. Additionally, stepper lithography is associated with high operational costs and infrastructural demands, limiting its economic scalability [61]. Therefore, alternative fabrication techniques such as nanoimprint lithography or replica molding have gained attention as more cost-effective options for large-scale nanoneedle production [24,61,62].

### 5.4. Biodegradable and Hydrogel-Forming Nanoneedles

Another category of nanoneedles includes biodegradable and hydrogel-forming nanoneedles [32,38]. Hydrogel-forming needle arrays are composed of cross-linked polymers or silicone, with occasional incorporation of metals. The polymers used in their fabrication, such as polyvinylpyrrolidone (PVP), are both biodegradable and biocompatible. These arrays can be produced in various patch sizes, effectively sterilized, and thoroughly removed from the skin after use [32].

This innovative technology leverages swellable or hydrogel-forming nanoneedles to deliver macromolecules. The loading capacity of these systems is not only limited by the physical space within the needles but also influenced by the cross-link density of the hydrogel. This technique expands the range of drugs that can be administered transdermally, enabling the delivery of treatments for conditions such as rheumatism, hypertension, insulin resistance, gene therapy, cancer, and vaccines [63]. When supported by an electrical supply, these systems can also be used for bolus and pulsatile drug delivery. The swelling of hydrogels upon contact with body fluids can cause the tips of the needles to break off intentionally, allowing them to remain at the application site for an extended period, facilitating sustained drug delivery [36,64,65]. Although this controlled swelling mechanism has not yet been investigated at the nanometric scale, it is conceivable that a similar approach could be applied to nanoneedles in future studies [32].

## 6. Fabrication of Nanoneedles

Nanoneedles are fabricated using various inorganic materials, such as metals and oxides, through two main strategies: the bottom-up and top-down approaches [35,66,67,68].

### 6.1. Bottom-Up Manufacturing Strategy of Nanoneedles

**Figure 3:**
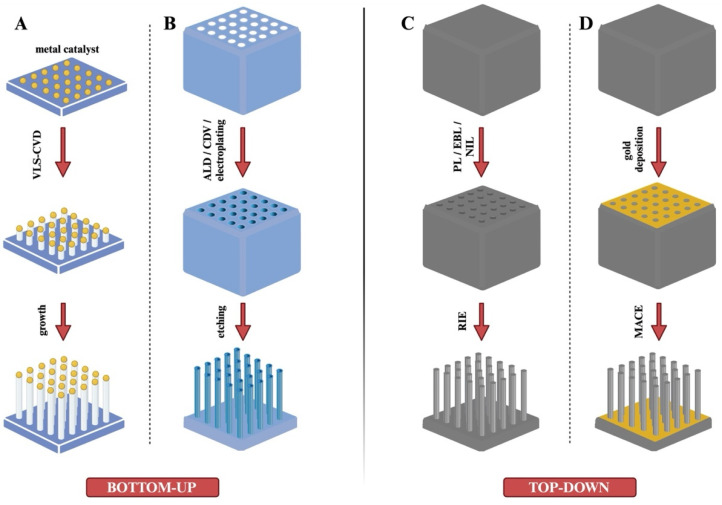
Fabrication of nanoneedles using different inorganic materials with bottom-up and top-down strategies. (**A**) Bottom-up strategy: Chemical vapor deposition (CVD) process. (**B**) Bottom-up strategy: ALD/CDV/electroplating method. (**C**) Top-down strategy: Reactive ion etching (RIE) method. (**D**) Top-down strategy: Metal-Assisted Chemical Etching (MACE) method.

The bottom-up strategy involves the accumulation of atoms or molecules along a specific path to build nanostructures. This process establishes a layer-by-layer configuration, where the creation of a high-aspect-ratio structure is essential (Figure 3A,B).

The accumulation of atoms or molecules represents the core of the bottom-up production methodology. Common techniques used in bottom-up fabrication include physical vapor deposition (PVD), chemical vapor deposition (CVD), and atomic layer deposition (ALD). While PVD has been explored for producing nanostructures, there is limited research on its application specifically in nanoneedle fabrication. As a result, most studies in this field have focused on CVD and ALD techniques, which have proven to be more effective for creating nanoneedles with the desired properties [24].

In the CVD process (Figure 3A), the substrate is exposed to one or more vacuum precursors, leading to the formation of nanoneedles through chemical reactions or decomposition on the substrate surface [69]. The production process typically involves three stages. First, the material to be deposited is gasified under an elevated temperature and pressure. Next, the gaseous reactants are introduced into the reaction chamber. Finally, chemical reactions occur between the reactants and the substrate, resulting in the deposition of a thin film on the substrate surface [24]. CVD is also referred to as the vapor–liquid–solid (VLS) process. It is commonly used to synthesize silicon dioxide (SiO_2_) and silicon nitride (Si_3_N_4_) films on silicon wafers, with potential applications in fabricating metallic materials. Both silicon and carbon can serve as materials for nanoneedles using this technique. CVD offers high efficiency and repeatability, but its main drawback is the high production cost, attributed to the expense of specialized equipment [24,68,69,70]. CVD can be used to fabricate both hollow and solid nanoneedles. The size, height, diameter, and density of nanoneedles can be customized by adjusting variables such as heating temperature, gas composition, reaction time, and catalyst size. However, certain limitations exist, such as the poor surface morphology due to high deposition temperatures and the constraints of layer-by-layer deposition [24]. Hollow nanoneedles can also be produced using CVD. Park et al. [23] reported the fabrication of hollow nanoneedles with highly ordered pores using anodized aluminum oxide (AAO) templates. In this process, a carbon layer was grown within the AAO structure. A gas mixture of acetylene (C_2_H_2_) and ammonia (NH_3_) was introduced into the CVD chamber, where argon gas was added to facilitate the pyrolysis of C_2_H_2_, leading to carbon deposition on the AAO templates. The final step involved removing the AAO template, yielding hollow nanoneedles. A similar fabrication process was reported by Golshadi et al. [71,72].

An alternative CVD technique used in nanoneedle production is metal–organic vapor phase epitaxy (MOVPE), also known as metal–organic chemical vapor deposition (MOCVD). In this process, metal–organic precursor complexes containing the target material undergo surface reactions, promoting nanoneedle growth. Unlike conventional CVD, MOVPE does not require a metal catalyst and operates at relatively lower temperatures, making it suitable for continuous and large-scale production. The thin-film deposition method known as ALD is based on the successive application of a gas-phase chemical process. Two precursors that react sequentially and self-limitingly with a substrate’s surface are used in the majority of ALD processes. Repeated exposure to distinct precursors can result in the gradual deposition of thin nanoparticles. Hollow nanoneedles have been fabricated using ALD, a uniform, low-temperature deposition technique [24].

### 6.2. Top-Down Strategy for Nanoneedles

The top-down strategy involves one-dimensional (1D) etching of substrates using advanced micro- and nanofabrication technologies. The fundamental technique in this approach is physical and chemical etching, which can be further divided into two categories: dry etching and wet etching. The etching process is carried out using high-energy rays, electron beams, laser beams, and ion beams. Additionally, nanoimprinting is utilized in the fabrication of nanoneedles [24].

#### 6.2.1. Metal-Assisted Chemical Etching (MACE)

Metal-assisted chemical etching (MACE) is a widely used wet etching method that employs gold or other noble metals as catalysts to initiate the etching process, resulting in the formation of nanoneedles. This technique is especially effective in producing silicon nanoneedles [73]. The MACE process begins with the deposition of a layer of metal particles (e.g., Au, Ag, Pt) on the silicon substrate (Figure 3D). The substrate is then immersed in an etching solution, typically containing hydrogen peroxide (H_2_O_2_) and hydrofluoric acid (HF). The metal particles act as catalysts, accelerating localized etching. As the etching progresses, the metal particles gradually sink into the silicon substrate, facilitating the formation of silicon nanoneedles. Chiappini et al. used MACE to fabricate porous silicon nanoneedles for the intracellular delivery of nucleic acids [36,64,67]. The dimensions of nanoneedles can be controlled by adjusting processing parameters such as the reaction time, temperature, and HF concentration. Compared to the bottom-up strategy, MACE offers higher efficiency and greater convenience. Its key advantages include the ability to produce nanoneedles on a large scale, simplicity, and cost-effectiveness [73]. However, several limitations persist. Controlling the distribution and size of the metal particles can be challenging, leading to variability in the size of the resulting nanoneedles [24,74]. Additionally, the use of etchants poses environmental risks.

MACE can be employed for applications such as drug and cell delivery. Various factors—such as the choice of metal, temperature, etchant composition, illumination, and the intrinsic properties of the silicon substrate (e.g., doping type, doping level, orientation)—can significantly influence the characteristics of the fabricated nanoneedles [73].

#### 6.2.2. Reactive Ion Etching (RIE)

Reactive ion etching (RIE) is a widely used dry etching technique (Figure 3C) that produces nanoneedles by bombarding the substrate with high-energy gas ions [75,76]. RIE is often used in conjunction with photolithography or deposition techniques to create masks, define substrate patterns, and determine the density, dimensions, and spacing of nanoneedle tips. During the process, a high-frequency electric field releases corrosive gases, generating high-energy ions for precise etching. RIE can be used to fabricate both solid and hollow nanoneedles. The technique provides high control over the production process, with the ability to adjust parameters such as geometry and structure. The advantages of RIE include excellent controllability, well-ordered structures, and high machining precision. However, it also has some drawbacks. High-energy gas ions can introduce side defects in nanoneedles, and the necessary microfabrication equipment is expensive. Additionally, nanoscale masks used in RIE are often produced by electron beam lithography, further increasing production costs [24].

#### 6.2.3. Focused Ion Beam Manufacturing (FIB)

The focused ion beam (FIB) manufacturing process uses an accelerated ion beam to selectively remove unwanted material from the substrate. FIB is a non-contact, high-energy fabrication method with excellent flexibility, making it suitable for processing a variety of materials. It is especially effective for creating high-aspect-ratio hollow nanoneedles with adjustable patterns, dimensions, shapes, and vertical side walls [24]. For instance, in a study by Zou et al. [77], pyramidal silicon nitride AFM tips were used to fabricate nanoneedles with a diameter of 200 nm and a length of 5–6 μm through FIB technology. Despite its precision and versatility, FIB has limitations, including a low processing speed and high equipment costs, which restrict its scalability for the large-scale production of hollow nanoneedles [24].

#### 6.2.4. Nanoimprinting

Nanoimprinting is a fabrication technique that creates nanoscale patterns through mechanical deformation [78]. In nanoneedle production, a negative template is often used to mold the desired pattern. The process typically begins with spin-coating a thin, print-resistant layer onto the substrate to act as a transfer medium. Patterns are then formed by pressing the negative template onto the substrate, deforming the resist layer. During the imprinting stage, the resist undergoes curing through heat or ultraviolet light, and the remaining layer is removed to finalize the pattern. Once the template is removed, the nanoneedles are produced. Nanoimprinting offers several advantages, including simplicity, cost-effectiveness, high efficiency, and high-resolution patterning. However, challenges remain, such as template overlay alignment, the occurrence of defects, template wear, and pattern fidelity. Nanoimprinting is commonly used in the field of biosensing [24].

While significant progress has been made in the fabrication of nanoneedles using various techniques, it is equally important to assess their translational potential. Parameters such as tip geometry, flexibility, and material biocompatibility directly affect critical clinical aspects like skin penetration, patient comfort, and safety. However, the number of studies evaluating these fabrication parameters in clinically relevant settings remains limited. Most current research is confined to in vitro or animal models, and further investigation is needed to validate the feasibility, scalability, and regulatory acceptability of these fabrication methods for clinical translation.

## 7. Important Parameters of Nanoneedles

- Insertion Force: The nanoneedles must be inserted with sufficient force to penetrate the skin effectively.
- Penetration: This parameter refers to the depth to which the nanoneedle system penetrates the *stratum corneum*. The depth of penetration determines the amount of drug delivered to the dermal and epidermal layers.
- Irritation: After inserting the nanoneedles, the skin is examined for signs of irritation, pruritus, or urticaria. Irritation tests are conducted using appropriate animal models, such as mice, rats, and rabbits.
- Drug Encapsulation and Content: This assessment determines the precise amount of therapeutic agents encapsulated within the nanoneedles.
- Needle Strength: Needle strength refers to the ability of the nanoneedles to effectively penetrate the *stratum corneum* without breaking.
- Drug Release: In Vitro Drug Release: This is performed by attaching the nanoneedle array to a glass slide and inverting it into a container with a suitable medium. In Vivo Drug Release: Experiments are conducted using animal models such as mice or rats, with aliquots taken at predetermined time points to quantify the active ingredients.
- Applicator Velocity: Applicator velocity refers to the speed and force with which the nanoneedles are applied to the skin. A higher applicator velocity improves the penetration efficiency.
- Tip Radius and Needle Length: Tip Radius: A smaller tip radius increases the penetrability of the nanoneedles. Larger tip radii reduce the ease of penetration. Needle Length: The length of the needle directly influences how deeply it can penetrate the skin. Transmission electron microscopy (TEM) and scanning electron microscopy (SEM) are used to measure these characteristics. Patch Thickness: The patch thickness can be measured using a digital caliper or a micrometer. The thickness affects patient compliance and the aesthetic appearance of the patch [32].

## 8. Toxicity of Nanoneedles

To date, toxicity studies of nanoneedles have been limited to in vitro cytotoxicity assessments. Research indicates that the cytotoxicity of nanoneedles is proportional to their diameter, as demonstrated in atomic force microscopy (AFM) studies using needles of various sizes. Additionally, the degree of membrane bulging and the insertion force directly influence cytotoxicity [79]. Intracellular fluid leakage during needle insertion is believed to be correlated with membrane bulging. A substantial amount of intracellular fluid may leak during insertion, potentially piercing the cell membrane and leading to cell death or significant cellular disruption. However, in cell culture studies, nanoneedle arrays with diameters below 200 nm did not cause notable cell death and had no adverse impact on cell metabolism or proliferation [47]. A similar trend has been observed with the internalization of nanorods. Nanoneedle systems with diameters between 200 and 300 nm have minimal effects on cell viability, with cells surviving on the silicon substrate for several days. The internalization of nanoneedles into cells does not appear to trigger immune responses or interfere with normal immune reactions to physiological stimuli. Gene expression profiling has revealed a distinctive pattern involving 300 immune-related genes [80]. Optimized nanoneedle systems, when force-inserted, can minimize adverse effects. Although nanoneedle insertion disrupts cell integrity, it does not induce toxicity. Cells cultured on nanoneedle arrays recognize the environment as a standard cell culture platform, maintaining their viability and normal functions [16].

Recent in vivo studies have contributed significantly to understanding the long-term toxicity and biocompatibility of nanoneedles. Chiappini et al. [36] demonstrated that biodegradable porous silicon nanoneedles can successfully deliver nucleic acids in vivo without inducing systemic toxicity or immune activation. Following intramuscular administration, the nanoneedles gradually dissolved under physiological conditions and completely degraded within 72 h, forming a temporary and non-disruptive interface with surrounding tissues. Moreover, histological analyses showed no signs of chronic inflammation, fibrosis, or necrosis at the application site. Similarly, another study [43] has reported that nanoneedle-treated tissues maintain a normal morphology and function over extended periods, suggesting favorable long-term tolerability. Moreover, Yin et al. [81] reported that polypyrrole-based nanoneedles, designed for combined chemo-photothermal therapy, exhibited a favorable biodistribution and were well tolerated after systemic administration in vivo, suggesting a promising biosafety profile without evident signs of systemic toxicity. These findings indicate that nanoneedles—especially those fabricated from biodegradable materials—are well tolerated in vivo and can serve as safe platforms for localized and sustained gene or drug delivery.

The sterilization, usage, and disposal processes of micro- and nanoneedle systems should be standardized to ensure safe use, particularly in home or clinical settings. This is not only important in terms of efficacy and stability but also in minimizing the risk of potential contamination or toxic residues. Swellable needles, in particular, are advantageous due to their ease of application and lower risk of leaving behind polymer residues compared to previous generations. However, in porous or hollow needles, the structures must be thoroughly cleaned after fabrication, as unpolymerized material residues may pose biological risks. Therefore, aspects such as the effectiveness of post-production cleaning protocols, the selection of suitable sterilization methods, and the assessment of potentially leachable contaminants should be more comprehensively evaluated from both clinical and regulatory perspectives [82,83,84].

## 9. Nanoneedles for Gene Delivery

In the near future, genetic medicines may revolutionize the treatment and even the cure of various diseases, including cancer and genetic disorders. Therapeutic interventions such as the expression of therapeutic proteins, gene repair or replacement, genome editing, and gene silencing are becoming increasingly available to patients [5].

However, the success of gene therapy is still constrained by several challenges. These include inefficient uptake of genetic materials by target cells, safety concerns, variability in effectiveness depending on the cell type and location, and difficulties in large-scale manufacturing. Furthermore, there are numerous biological effectors—such as plasmid DNA (pDNA), small interfering RNA (siRNA), self-amplifying RNA (saRNA), microRNA (miRNA), long non-coding RNA (lncRNA), and CRISPR RNA (crRNA)—each with different mechanisms of action. Consequently, new delivery methods are required to transport these materials effectively into cells and intracellular organelles to achieve the desired therapeutic outcomes. An ideal gene carrier system should be applicable to various cell types without compromising cell viability [45].

Nanoinjection using nanoneedles offers a biocompatible and reliable physical gene-delivery method. Nanoneedles are advantageous due to their ability to bypass cellular barriers with minimal invasiveness, their geometric flexibility, and their capacity to interact with multiple cells simultaneously. These tunable nanoneedles, which can carry pDNA and siRNA, enable the modulation of gene activity—either by inducing gene expression or silencing genes through RNA interference—or can be employed for genome editing in both in vivo and ex vivo applications [85,86,87]. Once nanoneedles penetrate the cells, various parameters, such as the cells’ tolerance to the applied force, delivery efficiency for DNA and RNA, toxicity, and effects on cell viability and proliferation, are actively investigated. Optimizing the geometry of nanoneedles and their interactions with cells is essential for enhancing therapeutic efficacy. Key factors influencing this interaction include the geometry, tip diameter, and cell-surface adhesion properties of the nanoneedles.

**Figure 4:**
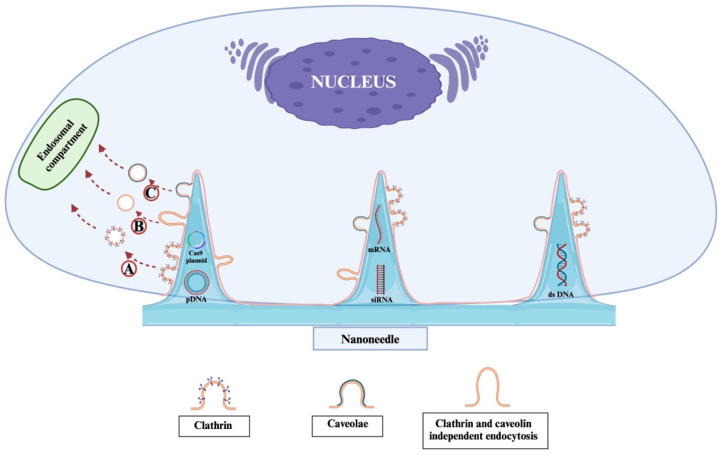
Schematic representation of the potential endocytic pathways involved in nanoneedle-mediated gene delivery. Upon interaction with the cell membrane, nanoneedles may trigger multiple types of endocytosis depending on cell type and nanoneedle surface chemistry. A. Clathrin-mediated endocytosis involves the formation of clathrin-coated pits that internalize the nanoneedle cargo via vesicular trafficking. B. Clathrin- and *caveolae*-independent endocytosis allows uptake through alternative, less characterized routes, possibly involving lipid rafts or macropinocytosis. C. *Caveolae*-mediated endocytosis utilizes flask-shaped plasma membrane invaginations rich in caveolin, facilitating cargo internalization without triggering significant immune activation. These pathways may operate in parallel or preferentially, and recent studies have shown an enrichment of *caveolae* and clathrin-coated pits at the nanoneedle–cell interface, supporting their role in gene cargo transport.

Nanoneedles achieve effective intracellular delivery by overcoming cellular barriers without causing significant damage to the cells. However, debates remain regarding the exact mechanisms through which nanoneedles facilitate cargo transport—whether by direct penetration of the cell membrane or by triggering other cellular uptake pathways [61,88,89]. Recent studies have observed an increased presence of *caveolae* and clathrin-coated pits at the cell–nanoneedle interface, suggesting that nanoneedles might employ different forms of endocytosis for cargo transport (Figure 4) [61,88]. However, it is important to note that the mechanism of nanoneedle-mediated delivery remains a subject of ongoing debate. While some studies support direct membrane penetration, others provide evidence for endocytic pathways, including clathrin- and *caveolae*-mediated uptake. These divergent observations may be attributed to differences in cell types, nanoneedle geometry, applied pressure, and surface functionalization. Therefore, while results such as those by Han et al. [31] and Chiappini et al. [90] are promising, further comparative studies are needed to clarify the dominant internalization mechanisms.

Higgins et al. [61] identified three key interactions between nanostructures and cells:

- The degree to which nanostructures are engulfed by the cell membrane varies.
- Under specific conditions, nanostructures can penetrate the membrane directly.
- Nanostructures can stimulate endocytosis.

To clarify the dominant uptake mechanism, future studies should incorporate structured experimental frameworks. These include the following:

- The use of pathway-specific endocytosis inhibitors such as clathrin-mediated, *caveolae*-mediated, or macropinocytosis to dissect internalization routes [91].
- Colocalization studies using fluorescently labeled endosomal and lysosomal markers to visualize intracellular trafficking [61,91].
- Real-time live-cell imaging to observe nanoneedle–cell interactions and cargo transport dynamics [92].
- And importantly, temperature-dependent uptake assays conducted at 4 °C and 37 °C to distinguish energy-dependent endocytosis from passive membrane penetration [93].

Integrating these methods into future mechanistic studies will be critical to uncover how nanoneedles mediate cellular entry and to inform the design of more effective delivery platforms.

Gopal et al. [91] demonstrated that silicon nanoneedles enhance siRNA uptake into human mesenchymal stem cells (MSCs) by modulating clathrin- and *caveolae*-mediated endocytosis, as well as macropinocytosis. While many siRNAs are trafficked through endolysosomal pathways, approximately 38% escape these routes, preserving their functionality in the cytosol.

Nanowires provide unique access to intracellular processes. Kim et al. [92] developed vertically aligned silicone nanowire (SiNW) arrays capable of transfecting plasmid DNA into multiple cells. They used confocal microscopy and SEM to demonstrate the physical interaction between nanowires and cells. The nanowires had 3–6 µm in length and had diameters of 30, 90, and 400 nm. Due to their small diameters and high aspect ratio, it was observed that SiNWs penetrated the cells within 1 h without a need to apply any force. Human embryonic kidney (HEK 293T) cells and mouse embryonic stem (mES) cells were cultured on a silicone (Si) substrate that had a vertically aligned SiNW array, and gene (*GFP* plasmid) transfer was demonstrated using the SiNW array. Despite the physical penetration of the nanowires, it was observed that the cells survived for one week and that the mES cells could differentiate on the SiNW array substrates.

Nucleic acids can be directly coated onto the surfaces of nanostructures. This allows them to interact directly with cells and modify gene expression. The cellular mechanism can be altered by producing the desired target protein from the transfected DNA, or the production of the desired protein can be suppressed by blocking mRNA translation with siRNA. Shalek et al. [45] investigated the ability of silicone nanowires to deliver biomolecules into cells with high efficiency without chemical modification or viral packaging. Using this method, they evaluated the effects of biological molecules (DNA, RNA, peptides, and proteins) that were easily delivered into almost any cell type. The nanowires (NWs) were coated with short aminosilane to enable non-covalent interactions with biomolecules. Through non-specific binding, siRNA was slowly released from the NW. They developed a scalable system capable of delivering siRNA, peptides, and proteins, both separately and together, to both primary and immortalized cells on a single substrate using vertically aligned silicone nanowires in a microarray format. It was shown that siRNAs delivered to cells via nanowires reduced transcript levels, peptides delivered inhibited apoptosis, and targeted proteins were transported to specific organelles.

Elnathan et al. [37] investigated the impact of the geometric parameters of DNA-coated vertically aligned silicone nanowire (VA-SiNW) arrays on cell behavior and transfection efficiency. They increased the contact area between the cell surface and the VA-SiNW. The VA-SiNW arrays enhanced DNA uptake by cells while maintaining cell viability. A high transfection efficiency was found in most cells treated with VA-SiNW, with medium heights (1.2–3.5 µm), small diameters (<400 nm), and densities between 0.6 and 1.0 NW µm^−2^. However, there were significant differences between the cell types. When optimal NW geometries were applied, the transfection efficiency was close to 90% for hDPSC and HEK293 cells, whereas it remained below 65% for HFF and HeLa cells throughout the study. It was observed that cell types with increased interaction with the VA-NW substrate, such as highly adhesive and widely spread cells, were more efficiently transfected with VA-NW arrays. The increase in transfection was reported to be a result of adhesive cells generally interacting with a greater number of NWs, thereby increasing the likelihood of penetration.

Chiappini et al. [90] demonstrated that the intracellular delivery of nucleic acids and the regulation of gene expression occurred using the nanoinjection strategy. They used biodegradable nanoneedles with metal-assisted chemical etching of silicone to load a *GFP*-expressing DNA plasmid and Cy-3 labeled siRNA simultaneously. It was shown that more than 90% of the co-delivered pDNA and siRNA reached the cytosol. The nucleic acids loaded onto the nanoneedles were reported to be released over more than 12–18 h. The biodegradable porous nanoneedles, which formed a temporary interface with cells, slowly dissolved under physiological conditions, losing their shape and completely degrading after 72 h. This study investigated the induction of neovascularization in muscle tissue using nanoneedles as gene carriers. Transfection of the *VEGF*-165 gene, which induces neovascularization, was performed using nanoneedles with a 50 nm diameter and 2 µm pitch, resulting in a six-fold increase in blood perfusion in the targeted muscle area. The newly formed blood vessels were functional, with blood flow rates similar to those of pre-existing vessels.

Nanoneedles are used to effectively deliver nucleic acids to hard-to-transfect cells such as primary neural, immune, stem, and corneal cells. They provide an effective therapeutic option by making the desired functional changes in the cell without disrupting the cell structure. Maurizi et al. [94] achieved effective gene silencing and protein knockdown by transfecting human corneal endothelial cells and explanted human corneas with porous silicone nanoneedles loaded with *p16*-targeting siRNA, showing approximately 73% and 79% gene silencing compared to the control, respectively. They used siRNA-loaded nanoneedles for the treatment of endothelial corneal dysfunction via a targeted RNAi strategy without causing any toxicity. It was demonstrated that the application of nanoneedles to primary cells resulted in successful interfacing, with localization in both the cytoplasm and nucleus.

The preparation of vertically aligned nanoneedles for gene transfection in cells using inorganic materials requires high-cost advanced nanofabrication machinery. The physicochemical properties of nanoneedles, such as topography, rigidity, porosity, and chemical composition, can be altered using different fabrication methods. All these factors affect the interface interactions of the nanoneedles with biological systems. Polymeric nanoneedles can be produced using cost-effective molding and nanoimprint lithography techniques. Yoh et al. [95] prepared polymeric nanoneedles using non-toxic, biocompatible polymers such as polystyrene, SU8, and polydimethylsiloxane (PDMS). They studied the interaction of polymeric nanoneedles with different stiffness levels but with the same topography with adherent and suspension mammalian cells by forming interfaces with the cells. The surface of polystyrene (PS) nanoneedles was functionalized with positively charged PDL to study the transfection of Cy5-tagged mRNAs encoding a *GFP* reporter into GPE86 (mouse embryonic fibroblasts) and L1.2 (mouse B) cells. The transfection efficiency with mRNA-loaded, PDL-coated PS nanoneedles was found to be higher in GPE86 cells (49.4%) compared to L1.2 cells (12%). They reported that the intracellular delivery by polystyrene nanoneedles occurs via a caveolin-1 and clathrin heavy chain-mediated endocytosis mechanism. Interestingly, while stiffness had a limited effect on overall mRNA delivery (26.8–33.2% across all materials), the combination of positive surface charge and strong adhesion appeared critical for high efficiency. These findings suggest that the surface charge and cell type-specific adhesion properties play a more dominant role in determining transfection efficiency than stiffness alone. Nevertheless, tuning both mechanical and chemical properties of polymeric nanoneedles offers a promising strategy for optimizing gene delivery across various cell types.

The most important challenge of CRISPR as a genome-editing tool is delivering it to the appropriate cells while minimizing off-target effects. In this regard, multi-purpose carriers can be employed. Each gene delivery system has its advantages and disadvantages, and delivery efficiency may vary between in vitro and in vivo settings. According to the current literature, viral vectors such as adeno-associated virus, adenovirus, and lentivirus; non-viral systems such as lipid-based nanoparticles, extracellular vesicles, polymeric and gold nanoparticles, mesoporous silica particles, and cell-membrane-derived vesicles; as well as physical methods like electroporation have all been explored. Additionally, the most effective gene-editing outcomes with minimal off-target activity have typically been achieved through ribonucleoprotein delivery [96,97,98]. Nanoneedles, particularly biodegradable ones, are promising due to their ability to deliver cargo directly into the nucleus, as well as their specificity and safety profile. Recently, nanoneedles have been studied for the delivery of CRISPR–Cas9 gene-editing tools. The route of administration of CRISPR systems requires careful evaluation, as systemic administration may result in unexpected adverse effects and off-target consequences due to the high volume of distribution. In terms of safety and accessibility, local routes of administration are more favorable in the transition to clinical applications. Yamagishi et al. [99] utilized 200 nm diameter silicone-based nanoneedles to facilitate the direct delivery of the sgRNA/Cas9 complex into the cellular compartment. They reported that *GFP* expression decreased by 32% with the delivery on HeLa cells of *GFP*-targeted sgRNA and Cas9 complex adsorbed on the hydrophobic surfaces of the nanoneedles, while target gene disruption was 15.4% with the delivery of *nestin*-targeted sgRNA and Cas9 complex to mouse breast cancer cells. Concurrently, CRISPR technology provides a rapid and sensitive approach to the assessment of adenosine triphosphate (ATP) levels in living cells, offering a valuable tool in the fields of cancer and biomedical research. Kim et al. [100] detected intracellular ATP levels in living cells in 30 min with nanoCRISPR, which they developed by functionalizing the surface of porous silicone nanoneedles with a Cas12 activator locked with ATP aptamer.

Some immune cells, such as primary Natural Killer (NK) cells, are very difficult to transfect with genes and proteins using conventional methods. Non-viral delivery systems, such as liposomes or nanoparticles, are internalized into cells via endocytic pathways, but immune cells have the ability to recognize these delivery systems as foreign and subsequently eliminate them. The introduction of genes into immune cells with viral vectors may present certain safety concerns and potential toxicity risks. The transfection of Cas9/Ribonucleoprotein (Cas9/RNP) complexes into cells, especially immune cells, presents significant challenges due to their considerable size. Li et al. [101] developed a safe and highly efficient vibration-assisted nanoneedle/microfluidic composite system for the transfection of Cas9/RNP complexes into NK-92 cells for the production of gene-modified NK cells by combining nanoneedles with microfluidic technology. Consequently, the transfection efficiency of Cas9/RNP complexes into NK-92 cells was enhanced to 98% without compromising cell viability.

The development of nanoneedles capable of delivering CRISPR/Cas9 tools through transdermal delivery holds promise for genome editing-based therapies, offering precise and safe localized treatment options.

Recent studies have demonstrated the feasibility of vehicle-free delivery strategies for microRNAs (miRNAs), which are single-stranded, short, non-coding nucleic acid molecules, offering a promising avenue for gene therapy without reliance on traditional carriers. For example, Abdelaal et al. developed a fully modified miRNA mimic that enabled an efficient cellular uptake and therapeutic efficacy without any delivery vehicle, showcasing the potential of chemically stabilized oligonucleotides in oncology [102]. Additionally, the same group demonstrated the efficient endosomal escape and functional intracellular delivery of chemically modified *miR-34a* mimics in vivo, as evidenced by durable target gene suppression and significant tumor regression [103]. These findings point toward a broader therapeutic scope for non-coding RNAs and suggest that nanoneedle-based transdermal systems, with their localized and minimally invasive delivery profile, may offer a synergistic platform to further enhance vehicle-free RNA delivery, particularly in targeted applications requiring spatial control and tissue specificity.

Throughout this section, we have discussed various genetic cargos—including siRNA, plasmid DNA, mRNA, and CRISPR/Cas9 complexes—and their delivery using different nanoneedle platforms. These systems differ not only in structure and material but also in their capacity to accommodate various cargo sizes while maintaining delivery efficiency. However, despite the growing interest in nanoneedle-mediated delivery systems, systematic comparisons of their cargo size limitations and payload capacities are largely absent from the literature, highlighting a significant gap that future studies should aim to address.

Notably, nanoneedle systems are beginning to enter clinical evaluation. For instance, clinical trial NCT04492943 is investigating a silicon nanoneedle platform for intradermal mRNA delivery, marking an important step toward clinical translation. In another clinical study numbered NCT05853107, the AuTNA I system, consisting of titanium dioxide nanowire arrays (nanoneedle-like structures) coated with gold nanoparticles, was subretinally implanted to replace damaged photoreceptors in patients with retinitis pigmentosa. In the clinical study numbered NCT04390490, the sensitivity, specificity, and efficacy of a photoelectrochemical immunosensor using silicon nanowire structures combined with graphene quantum dots in the early diagnosis of acute myocardial infarction were evaluated. However, compared to nanoneedle systems, FDA-approved nanoneedle-based delivery platforms are still lacking, and most nanoneedle applications remain in preclinical or proof-of-concept phases. This underscores the need for continued research into their long-term safety, scalability, and regulatory pathways.

## 10. Conclusions

The integration of nanotechnology with nanoneedle strategies has recently enabled the effective delivery of genetic molecules for gene therapy. Nanoneedles offer a novel, non-invasive approach to transdermal drug delivery, providing a painless alternative to traditional methods. Key advantages include a simple delivery mechanism, precise dosing, enhanced bioavailability, and improved drug stability. Gene therapy is a groundbreaking method for treating and preventing diseases by using genetic materials such as DNA, mRNA, siRNA, and genome-editing enzymes. However, challenges remain in the effective delivery of these materials due to their enzymatic sensitivity, difficulty in cellular uptake, and the risk of off-target effects. Transdermally applied nanoneedles present a promising solution to overcome these challenges, facilitating the efficient delivery of genetic molecules directly into target cells while minimizing invasiveness and improving therapeutic outcomes. Nevertheless, translating nanoneedle technology from bench to bedside remains a significant challenge. Issues such as the scalability of fabrication methods, regulatory approval pathways, device reproducibility, and long-term safety assessments must be addressed before clinical implementation can be realized. While preclinical data are promising, further interdisciplinary research and standardization are essential to ensure the safe and effective clinical adoption of nanoneedle-based gene delivery systems. In parallel, the establishment of standardized performance metrics—such as delivery efficiency, penetration depth, cell viability, degradation rate, and immune response—is crucial to enable reproducible evaluation and facilitate comparison across studies.

These promising clinical advances highlight the translational potential of nanoneedle systems, yet several key steps remain to ensure their widespread clinical adoption. Various critical stages need to be completed to ensure the successful transition of nanoneedle-based systems into the clinical setting. These include the initiation of early-stage clinical trials, the development of standardized protocols for sensitive applications such as the delivery of genetic material, and the establishment of large-scale, reproducible, and regulation-compliant manufacturing processes. Implementing these steps will enable nanoneedle technologies to move beyond the research phase and be integrated into real-world clinical applications, thereby accelerating translational progress in the field.

## Figures and Tables

**Table 1:** Microneedle vs. nanoneedle [16,21,22,23,24,25,26,27,28,29].

	Microneedles (MNs)	Nanoneedles (NNs)
**Definition**	- MNs range in length from 150 to 1500 μm, with a width between 50 and 250 μm, and a diameter of 1 to 25 μm, enabling the formation of micron-sized pores in the skin. - The efficiency of transdermal drug delivery using MNs is influenced by the rate of drug transport across the epidermis.	- NNs are defined by their high-aspect-ratio design, with diameters at the nanoscale. - Thin one-dimensional (1D) nanostructures can penetrate the cell membrane by applying highly localized stress through sharp nanofeatures with diameters ranging from 1 to 100 nm. These systems have applications in both cell detection and drug delivery. - The term “nanoneedles” encompasses a variety of nanomaterials, including nanowires, nanosheets, nanocones, nanorods, and nanotubes. - There are two primary types of NNs: Vertical high-aspect-ratio arrays—These structures are supported by a substrate or bottom layer. External single nanoneedles—These individual needles are designed to establish direct contact with cells and tissues. Single nanoneedles, most often in the form of nanowires, are typically operated using atomic force microscopy (AFM) or micromanipulators.
**Size**	- MNs are micron-sized needles, available in various materials and shapes. Their length ranges from 25 to 2000 μm, corresponding to the typical thickness of the epidermis, including the *stratum corneum* (10–20 μm). The tip radius of MNs falls between 1 and 25 μm, ensuring effective penetration into the skin.	- The needles measured 5–7 μm in height, with a tip diameter of less than 50–200 nm and were arranged in a square lattice with a pitch of 2 μm.
**Superiority over other forms**	- The delivery and sampling methods are pain-free, minimally invasive, easy to dispose of, and designed for self-administration. - The needles must be long enough to penetrate the *stratum corneum* but short enough to avoid reaching nerve endings, ensuring minimal discomfort. - Compared to oral and topical administration, this approach is more effective. - This novel strategy enhances the effectiveness of conventional transcutaneous drug delivery.	- Since NNs are significantly smaller than MNs, they are less likely to cause skin damage, even with repeated use. - NNs, due to their nanoscale size, are frequently employed to penetrate the cell membrane, allowing for nearly invisible and direct delivery into the cytoplasm. - Unlike conventional delivery systems, NNs reduce the risk of carrier-induced toxicity within cells and prevent drug degradation caused by endosomal entrapment. - This novel strategy enhances conventional transcutaneous drug delivery. While NNs operate on the same principles as MNs, their smaller nanometer-sized structures enable a superior performance. - NNs can pierce the cell membrane, facilitating the introduction of biological molecules into cells. - The penetration process minimally disrupts the cells, allowing them to remain healthy post-treatment.
**Design**	- MNs are available in various forms, including solid, coated, and hollow types, as well as dissolving, hydrogel-forming, and bioresponsive designs. - These specialized designs enhance the drug-loading capacity, improve bioavailability, and increase adhesion. They can be adapted for application to a range of targeted areas, from the skin to the eyes.	- NNs are available in various types, including solid, coated, hollow, biodegradable, and porous forms. Solid NNs: Capable of entering the cytosol by piercing cell membranes. Coated NNs: Used to analyze cytoskeletal composition. Porous NNs: Ideal for the sustained delivery of concentrated cargoes due to their large surface area. Biodegradable NNs: Suitable for sustained drug release as they gradually degrade over time. Hollow NNs: Used to inject theranostic materials directly into cells.
**Fabrication**	- The fabrication of MNs is based on traditional techniques such as photolithographic procedures, laser cutting, metal electroplating, metal electropolishing, silicon etching, and micromolding. - Deposition processes used in MN fabrication are categorized into two types: chemical and physical, depending on the nature of the process. Chemical deposition: Includes techniques such as plating, spin coating, and chemical vapor deposition (CVD). Physical deposition: Encompasses methods like thermal evaporation, laser ablation, sputtering and ion plating, molecular beam epitaxy, and cluster-beam deposition.	- Fabrication techniques at the nanometer scale undergo extensive experimental investigations. - There are two primary strategies for fabricating NNs: top-down and bottom-up approaches. Bottom-up fabrication techniques: These methods focus on building structures atom by atom or molecule by molecule. Common techniques include physical vapor deposition (PVD), atomic-layer deposition (ALD), and chemical vapor deposition (CVD). Top-down fabrication techniques: This approach utilizes advanced nano-manufacturing technologies to etch substrates into one-dimensional (1D) NNs. The essential processes for this strategy include both physical and chemical etching methods.
**Materials**	- Fabricated with metals, polymers, silicone, silicone dioxide, ceramic, glass, and other materials.	- Fabricated with metals, polymers, silicone, and silicone dioxide.
**Drug delivery**	- MNs enable the direct and controlled delivery of small molecules, macromolecules, vaccines, and nucleic acids into the epidermis. - Frequent dosing is not required, as drugs can quickly diffuse for localized delivery within the skin or achieve systemic distribution through dermal capillaries. - MNs penetrate the *stratum corneum* barrier, delivering drugs to the epidermis and superficial dermis. - By encapsulating drugs directly within poly(lactic-co-glycolic acid) (PLGA) MNs, drug-release kinetics can be controlled over periods ranging from hours to months. - The release of loaded drugs is governed by three primary mechanisms: diffusion, degradation, and triggered release, all of which depend on the size of the drug and the properties of the polymer mesh.	- NNs represent a novel technique that enables the controlled release of drugs and bioactive molecules. - NNs offer a viable option for the long-term, non-immunogenic transport of molecules into whole tissues in vivo as well as isolated cells in vitro. - They have applications in tissue regeneration, gene therapy, and cancer treatment. - NNs facilitate the intracellular delivery of siRNA and DNA, as well as peptides and cell probes, which are used to transport biomolecules such as genes, drugs, and proteins into cells. - This delivery route ensures that the drug reaches the targeted area, thereby minimizing side effects.
**Applications**	- MNs can be utilized for both transdermal drug delivery and transdermal sensing. - They have applications in various areas, including the treatment of topical lesions, blood glucose control, cancer, obesity, migraines, and Alzheimer’s disease. Additionally, MNs are used for contraception, osteoporosis management, vaccine delivery, ocular disease, and cosmetic purposes.	- The use of NNs in clinical applications has expanded significantly, with extensive research focused on optimizing them for molecular delivery, stem cell therapy, regenerative medicine, and disease monitoring, diagnosis, and treatment—including conditions such as Parkinson’s disease, depression, and obsessive–compulsive disorder. - NNs have applications in gene transfection, cell manipulation, biosensing, and the management of diabetes, rheumatoid arthritis, inflammation, cancer, and hypertension. They are also utilized for vaccine delivery, cosmetic procedures, drug targeting, and other therapeutic purposes. - Additionally, NNs help minimize drug accumulation at non-target sites, reducing potential side effects.
**Dynamic delivery strategies/Triggered release**	- Stimuli–response–exogenous stimulant–heat/electricity. - Stimuli–response–endogenous stimulant–glucose.	- Improvement in electricity. - Improvement in magnetic force. - Stimuli-responsive system. - A new strategy for the application of personalized treatment, where the therapeutic index is maximized by both the smaller dosage needed and its targeted delivery.
**Problems**	- Skin sensitivity or allergies may cause skin inflammation. - High dosages can also result in localized inflammation. - Applying MNs to the skin presents challenges, as the tips of the needles may break off and become embedded beneath the skin.	- Although porous needles may appear more brittle than their solid counterparts, their porosity can be controlled, and their mechanical properties enhanced. - There is conflicting evidence suggesting that NNs may closely associate with the cell membrane without fully penetrating it.
**Toxicity**	- MNs demonstrated low off-target toxicity, improved tumor targeting, and decreased toxic side effects.	- The toxicity of NNs has primarily been studied in vitro, with research limited to investigations of cytotoxicity. - Studies involving needles of varying diameters have demonstrated that cytotoxicity decreases as the diameter decreases, correlating with the degree of membrane swelling and the force required for insertion. - Minimal swelling occurs with reduced insertion force, resulting in little to no leakage, and cells are more likely to avoid damage from NN penetration. - NNs with diameters around 200 nm or smaller have minimal impact on cell viability and proliferation following insertion. In contrast, diameters exceeding 400 nm result in the death of a substantial majority of cells interacting with the NNs.
**Approved products**	- Early in 2020, the Qtrypta^®^ new drug application was approved by the FDA. - Several MN systems are FDA-approved or in clinical trials.	- NN systems are in clinical trials (NCT04492943, NCT05853107, and NCT04390490). - NanoMosaic LLC is licensed for early disease detection. - Knowledge about the dynamics and localization of NNs is still limited.

**Table 2:** Types of nanoneedles.

Type	Material	Characteristics	Use	References
Solid	- Metal - Silicone - Polymer	- Adequate mechanical strength - Sharper tip - Drug loading via physisorption	- Drug delivery - Access to cytosol	[16,39]
Hollow	- Silicone	- Empty shape - To be filled with liquid formulation - Ability to control drug release - Large dose/amount of drug	- Diagnosis - Inject theranostics into cells - Vaccine delivery	[16,32,40,41]
Coated	- Silicone	- Carry lower amount of drug - Delivery of DNA and protein - Coated with enzymes, antigens, etc.	- Drug delivery - Vaccine delivery - Detection of cytoskeletal components	[16,32,42]
Biodegradable	- Biodegradable material - Silicone	- Slowly degrade - Porosity could be tailored according to degradation time	- Sustained-release drug delivery	[36]
Porous	- Silicone	- High surface area - Large reservoir - Electrostatic loading method - Slowly release	- Nucleic acid delivery - Cancer therapy - Sustained-release drug delivery	[16,32,43,44]

## Data Availability

The contributions presented in this study are included in the article.

## Abbreviations

The following abbreviations are used in this manuscript:

FDA	U.S. Food and Drug Administration
ZFNs	Zinc Finger Nucleases
TALENs	Transcription Activator-Like Effector Nucleases
AFM	atomic force microscopy
VLS	vapor–liquid–solid
FIB	focused ion beam
APTES	3-(aminopropyl) triethoxysilane
pDNA	plasmid DNA
siRNA	small interfering RNA
saRNA	self-amplifying RNA
miRNA	microRNA
lncRNA	long non-coding RNA
crRNA	CRISPR RNA
MSCs	mesenchymal stem cells
SiNW	silicone nanowire
mES	mouse embryonic stem
VA-SiNW	vertically aligned silicone nanowire
